# FERONIA and reactive oxygen species: regulators in the self-incompatibility response and in interspecific pollination

**DOI:** 10.1186/s43897-023-00058-z

**Published:** 2023-04-28

**Authors:** Zihan Song, Sheng Zhong, Li-Jia Qu

**Affiliations:** State Key Laboratory for Protein and Plant Gene Research, Peking-Tsinghua Center for Life Sciences, New Cornerstone Science Laboratory, College of Life Sciences, Peking University, Beijing, 100871 P. R. China

## Abstract

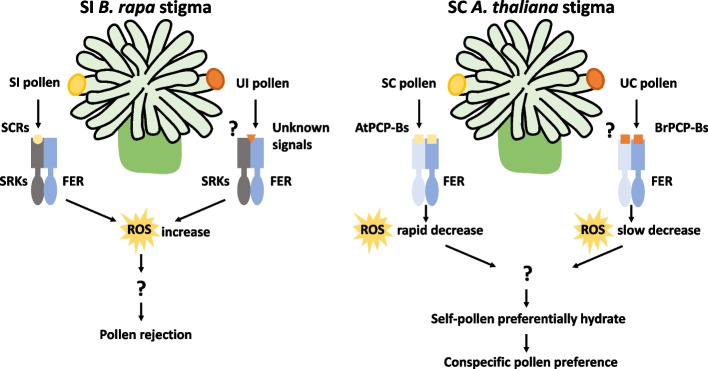

In plants, the stigma represents the initial site of pollen-pistil communication and serves as a crucial location for rejection of incompatible pollen. In self-compatible (SC) plants, such as *Arabidopsis thaliana* from the Brassicaceae family, pollen coat protein B (PCP-B) peptides were reported to compete with stigmatic RAPID ALKALINIZATION FACTOR 33 (RALF33) peptides for binding to the FERONIA/ANJEA (FER/ANJ) receptors in the stigma to promote pollen hydration via inhibition of reactive oxygen species (ROS) production (Liu et al. 2021). In self-incompatible (SI) members of the Brassicaceae, however, a different protein, the S-locus cysteine-rich protein (SCR), in the pollen coat was reported to bind to the stigmatic S-locus receptor kinase (SRK) at the same S-locus to trigger the SI response (Franklin-Tong 2002). A recent study showed that, in the SI plant *Brassica rapa*, signaling mediated by FER, a *Catharanthus roseus* receptor-like kinase-like 1 (CrRLKL1) family receptor (Wang et al. 2022), controls self-pollen rejection through increase in the ROS content of the stigma (Zhang et al. 2021). A long-standing unresolved question has been whether the SI system is involved in the control of interspecific isolation in SI plants. A study conducted in *A. thaliana* revealed that a stigmatic transmembrane protein, STIGMATIC PRIVACY 1 (SPRI1), facilitates the selection of interspecific pollen in a SI-independent manner (Fujii et al. 2019). Interestingly, hybridization between different plant species typically follows the SC × SI rule, wherein SC species tend to accept the pollen of SI species (termed unilateral compatibility, UC), whereas pollen is typically rejected in the reciprocal cross (termed unilateral incompatibility, UI). However, the mechanism of the SC × SI rule is unclear.

Recently, in a paper published in Nature, Huang et al. (2023) reported that, in the SI plant *B. rapa*, the SRK receptors not only reject SI pollen, but also reject interspecific pollen. To achieve this, the SCR from self-pollen or unidentified signals from interspecific pollen promote SRK to recruit FER and increase the ROS content in the stigma. If BrSRK or BrFER1 is silenced or the ROS content is reduced in the stigma, the acceptance of interspecific pollen and the efficiency of obtaining interspecific hybrid embryos are improved.

This paper demonstrated that, on SI stigmas, SRK controls rejection of UI pollen through FER-ROS signaling. The supporting evidence was as follows. 1. The expression level of SRK increases as the stigma of *B. rapa* matures, resulting in an increasingly stringent rejection of SI and UI pollen and, in both cases, ROS contents are increased in the stigma. Sequestration of ROS with sodium salicylate resulted in partial disruption of the rejection of UI pollen, allowing the penetration of *B. oleracea* pollen tubes into the mature stigma and *Barbarea vulgaris* pollen tubes into *B. rapa* stigma at the bud stage. 2. Knock down of BrSRK46 or expression of a transmembrane domain-deleted SRK (BrSRK^Δ^™) resulted in less stringent rejection of UI pollen, consistent with the observed reduction in ROS content. 3. When SI modules of *A. halleri* were expressed in transgenic *A. thaliana* plants, acceptance of interspecific pollen by the *A. thaliana* stigmas was inhibited, whereas the stigmatic ROS content rapidly increased. These findings suggest that increase in SRK/SRK-dependent stigmatic ROS are associated with UI pollen rejection, similar to the SI response.

Based on the reported role of CrRLK1L receptors in the regulation of ROS production, the authors of this paper aimed to determine whether the stigma-expressed BrFER1 and BrANJ1 were also involved in the UI response. They found that silencing BrFER1 or BrANJ1 resulted in inhibition of the ROS increase, and compromised SI and UI pollen rejection, which was consistent with the phenotype observed in plants with *BrRBOHF* silenced by antisense technology (AS-BrRBOHF). In addition, BrSRK46 interaction with BrFER1 was enhanced by protein extracts from SI and UI pollen as well as BrSCR46. These results suggest that the SI-determinant SCR from SI pollen and unknown signals from UI pollen promote the recruitment of FER by SRK, thereby activating FER-dependent ROS production to reject incompatible pollen. On the basis of this mechanism, reduction of ROS contents or disruption of Br-SRK/BrFER-to-BrRBOH signaling by AS-oligodeoxyribonucleotides in *B. rapa* stigmas led to the promotion of interspecific hybridization and the generation of hybrid embryos.

In the SC plant *A. thaliana*, the study showed that, although pollen from SI species could be accepted by the stigma, the resulting pollen tubes were much shorter than those of self pollen. The authors found that FER was also involved in this process, because *B. rapa* pollen hydrated more rapidly in *fer-4* stigmas and the tubes were much longer than those in wild-type *A. thaliana* stigmas. In interspecific pollination, reduction of the stigmatic ROS content was slower, indicating that FER–ROS functions in the discrimination against interspecific pollen to maintain species integrity. Given that PCP-Bs were reported to bind to FER and reduce the ROS content to promote pollen hydration, the authors further found that conspecific PCP-Bs had higher binding efficiency with the extracellular domain of FER, which may favor conspecific pollen for fertilization and thus serve as a potential interspecific barrier. Thus, this report shows that the SI system is capable of serving as an interspecific barrier, shedding light on the role of SRK in interspecific isolation and that of FER–ROS signaling in the establishment of reproductive barriers in SI and possibly SC plants (Fig. 1).

**Fig. 1:**
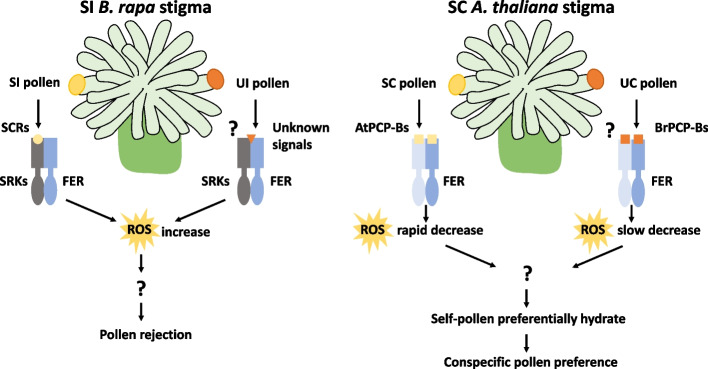
FER-ROS signaling is involved in the establishment of reproductive barriers in SI and possibly SC plants

## Future perspectives

Although all components in the SI response, such as SCR and SRK, and the FER–PCP-B–ROP2–RBOH cascade, have been previously described in intraspecific pollination, the authors have identified novel functions of these components in interspecific hybridization. This comprehensive study points to the potential utility of the ROS signaling system in agriculture to promote interspecific hybridization among SI plants. It also raises many questions that require clarification in the future. For instance, protein extracts from UI pollen promoted the interaction between BrSRK and BrFER, suggesting the existence of unknown SRK–FER-dependent signals in UI pollen. Silencing of BrSRK or reduction of the ROS content was insufficient to allow mature *B. rapa* stigmas to accept pollen from more distantly related heterogeneric species (*e.g.*, *B. vulgaris*), indicating that reproductive isolation is more stringent for more distantly related species and that there must be additional SRK-independent barriers. Furthermore, how do high contents of ROS lead to the rejection of undesirable pollen, through toxifying the pollen or by changing the status of the stigma? How does a reduced ROS content promote pollen hydration? Is the incompatible pollen completely unable to hydrate on SI stigmas? These are important questions that need to be investigated in the future. With regard to the role of ROS, intriguingly, the authors investigated whether the FER-ROS signaling pathway is associated with the known SRK signaling, and found that the silencing of BrARC1 did not inhibit SI- and UI-triggered ROS increase, but compromised the rejection of SI and UI pollen. This result suggests that a high content of ROS may not be essential for the rejection of undesirable pollen, nor does it have a toxic effect on the pollen. Similarly, in SC stigmas, the stigmatic barrier seems much weaker in the *fer-4* mutant than in the ROS-production-defective *rbohd* mutant, raising the possibility that, in addition to ROS, there must be other downstream product(s) of FER-dependent signaling that favor conspecific pollen in SC plants.

In this paper, the authors, by manipulating the elements in the UI response, namely BrSRK, BrFER, BrRBOH, ROS, or nitric oxide (NO) levels, were able to obtain interspecific hybrid embryos in SI plants. Despite the low efficiency, this approach provides the possibility of introducing desirable traits from distantly related species into crops via sexual reproduction. As reported previously, there are additional pre-zygotic reproductive barriers during pollen-pistil interaction in SC plants (Zhong and Qu 2019; Hater et al. 2020), such as pollen tube guidance (Zhong et al. 2019) and pollen tube reception (Escobar-Restrepo et al. 2007; Zhong et al. 2022). Given the limited understanding of interspecific/intergeneric isolation, dissecting the molecular mechanisms of other pre-zygotic/post-zygotic interspecific/intergeneric barriers would lay the foundation for distant hybridization of crops to secure food production for humans in the future.

## Data Availability

Not applicable.
